# Conjugative IncHI2/HI2A plasmids harbouring *mcr-9* in colistin-susceptible *Escherichia coli* isolated from diseased pigs in Japan

**DOI:** 10.1099/acmi.0.000454

**Published:** 2022-11-28

**Authors:** Akira Fukuda, Hitomi Nakano, Yasuhiko Suzuki, Chie Nakajima, Masaru Usui

**Affiliations:** ^1^​ Laboratory of Food Microbiology and Food Safety, School of Veterinary Medicine, Rakuno Gakuen University, Ebetsu, Japan; ^2^​ Hokkaido University International Institute for Zoonosis Control, Division of Bioresources, Sapporo, Hokkaido, Japan

**Keywords:** colistin, *Escherichia coli*, *mcr*, plasmid

## Abstract

Colistin is a last resort antimicrobial used for the treatment of gram-negative bacterial infections. Plasmid-mediated colistin resistance (*mcr*) genes are a cause of global concern, and, thus far, *mcr-1–10* have been identified. In a previous study, we screened *mcr-1–5* in *Escherichia coli* derived from diseased pigs in Japan and reported a high prevalence of *mcr-1*, *-3* and *-5*. However, the previous report on the prevalence of *mcr* genes was inaccurate. In the present study, we aimed to clarify the prevalence of all reported variants of *mcr* in *E. coli* derived from the diseased pigs, which were previously screened for *mcr-1–5*. Additionally, we also characterized the *mcr-9*-positive *E. coli *, which was detected in this study. We screened *mcr* in 120 *E. coli* strains from diseased pigs and *mcr*-positive *E. coli* and an *mcr*-carrying plasmid were also characterized. One *mcr-9*-positive colistin-susceptible *E. coli* strain was detected (0.8 %). Plasmid-mediated *mcr-9* was transferred to *E. coli* ML4909 as the recipient strain, and it was located on IncHI2/HI2A plasmid p387_L with other antimicrobial resistance genes (ARGs). The region harbouring ARGs including *mcr-9,* was similar to that on the *Klebsiella pneumoniae* chromosome harbouring *mcr-9* isolated in Japan. *mcr-3*, *-5* and *-9* were detected (4.2 %) in colistin-susceptible strains. *mcr-9* was found to be disseminated via the plasmid IncHI2/HI2A p387_L and transferred and inserted into chromosomes via a transposon. Our results suggest that *mcr* genes should be monitored regularly, regardless of their susceptibility to colistin.

## Introduction

The plasmid-mediated colistin resistance (*mcr-1*) gene was first reported in 2015 and has since been identified in various bacterial species worldwide [1]. Colistin is used as a last resort antimicrobial for the treatment of multidrug-resistant gram-negative bacterial infections in clinical settings for humans [2]. In livestock, colistin has been used as a feed additive and also for the treatment of gram-negative bacterial gastrointestinal infections. *mcr* genes are frequently detected in swine-pathogenic *Escherichia coli* [3, 4]. Following reports of the prevalence of *mcr* genes in livestock, several governments, including Japan, have restricted or banned the usage of colistin in livestock [5].

To date, ten *mcr* genes (*mcr-1–10*) have been reported, with the last variant (*mcr-10*) reported in 2020 [6]. Globally, *mcr-1* is the most frequently detected gene in colistin-resistant bacteria, followed by *mcr-3* and *mcr-5* [5, 7]. This trend has also been observed in Japan [3]. The effect of each *mcr* variant on colistin resistance is variable, with some *mcr* variants not conferring resistance to colistin in certain bacterial species and being silently disseminated [2, 8, 9]. However, overexpression of these genes can confer resistance to colistin [10]. Therefore, it is necessary to clarify the prevalence of *mcr* genes in both colistin-susceptible and colistin-resistant strains.

Following restrictions and bans on colistin usage in livestock, colistin resistance and *mcr* positivity have been reduced but not eliminated; a stable colistin resistance rate has been maintained [11]. Application of various types of antimicrobials can lead to selective pressure for maintaining *mcr* genes because of the co-localization of other antimicrobial resistance genes (ARGs) on plasmids harbouring *mcr* genes [12]. Specific types of mobile elements, including plasmids, are associated with the dissemination of specific ARGs [7]. Therefore, analysis of *mcr*-carrying plasmids is important to control the dissemination and maintenance of antimicrobial resistance.

We previously surveyed *mcr-1–5* in diseased pigs in Japan and reported a high prevalence of *mcr-1*, *-3* and *-5* [3]. In the present study, we additionally screened for *mcr-6–10* to clarify the prevalence and characteristics of these genes in the previously tested *E. coli* strains isolated from diseased pigs in Japan through retrospective analysis. We also characterized a newly identified *mcr-9*-positive *E. coli* strain.

## Methods

### Detection of *mcr-6–10* in *E. coli* from diseased pigs

In our previous study, *E. coli* strains (*n*=120) were isolated from pigs, inhabiting 40 farms in Japan, with post-weaning diarrhoea. Of these strains, 73 (60.8 %) were resistant to colistin and 75 (62.5 %) harboured *mcr-1*, *-3* and/or *-5* (Table 1) [3]. In the present study, we screened for *mcr-6–10* in these *E. coli* strains using PCR, as described previously [13, 14].

**Table 1.** Susceptibility to colistin and the presence of *mcr* genes

*Mcr* gene*	Colistin		
	Resistance	Susceptible	Reference
	(*n*=73)	(*n*=47)	
*mcr-9*	0	1	this study
*mcr-1*	31	0	[3]
*mcr-3*	8	2	
*mcr-5*	27	2	
*mcr-1* and *-5*	5	0	
nd	2	42	

*mcr-1–5 were screened in the previous study [3].

*mcr-2*, *-4*, *-6*, *-7*, *-8* and *-10* were not detected.

ND, not detected.

### Antimicrobial susceptibility testing

MICs were determined for the newly detected *mcr*-positive *E. coli* strain Ec387 using the micro broth dilution method according to the Clinical Laboratory Standards Institute (CLSI) guidelines [15]. Antimicrobial susceptibility was tested for the following antimicrobial agents: colistin, ampicillin, cefazolin, cefotaxime, gentamicin, kanamycin, tetracycline, nalidixic acid, ciprofloxacin, chloramphenicol and trimethoprim (all obtained from Sigma-Aldrich, St. Louis, MO, USA). Resistance breakpoints were defined according to the CLSI guidelines for Enterobacterales. *E. coli* ATCC25922 and *Pseudomonas aeruginosa* ATCC27853 were used for quality control.

### Conjugation experiments

Transferability of the newly detected *mcr* was tested using filter-mating methods with slight modification [16]. Briefly, the rifampicin-resistant K-12 ML4909 *E. coli* strain was used as the recipient strain, and mating was conducted at 37 °C. Transconjugants were selected on trypticase soy agar supplemented with 50 mg l^−1^ rifampicin (Sigma-Aldrich) and 1 mg l^−1^ colistin, 64 mg l^−1^ ampicillin, 32 mg l^−1^ kanamycin, 16 mg l^−1^ tetracycline, 32 mg l^−1^ chloramphenicol or 16 mg l^−1^ trimethoprim. Transconjugants were then tested for susceptibility to antimicrobials and the presence of *mcr* as described above.

### Whole-genome sequencing and subsequent bioinformatics analysis

We performed whole-genome sequencing of the *E. coli* strain Ec387. Genomic DNA for short-read sequencing was extracted using QIAquick PCR Purification Kit (QIAGEN, Hilden, Germany). The contig was mapped with 150 base pair (bp) paired-end reads obtained using Nextera XT and HiSeq sequencing platforms (Illumina, San Diego, CA, USA). Illumina reads were assembled *de novo* using spades 3.15.3 (https://github.com/ablab/spades) with default parameters. Genomic and plasmid DNA for long-read sequencing was extracted using Genomic-tip 20 G^−1^ and Genomic DNA Buffer Set (QIAGEN). The library was prepared using Rapid Barcoding Sequencing kit SQK-RBK004 (Oxford Nanopore Technologies, Oxford, UK) according to the manufacturer’s protocol. All bead washing steps were performed using AMPure XP beads (Beckman Coulter, Brea, CA, USA). Sequencing was performed on MinION with a FLO-MIN-106 R9.4 flow cell (Oxford Nanopore Technologies) using MinKNOW software with a 48 h run time and no alterations to any voltage scripts. Long-read sequencing reads were demultiplexed using Porechop v0.2.4 (https://github.com/rrwick/Porechop), and the reads were adaptor-trimmed and quality-filtered using NanoFilt (*Q* score, 9; minimum length, 1 000 bp). The reads were error-corrected using short-read sequencing reads with LoRDEC v0.9 software following default parameters [17]. *De novo* assembly was performed using Flye v2.9 with default parameters using error-corrected long-read sequencing reads [18]. Assembled contigs were error-corrected twice using short-read sequencing reads with Pilon v1.24 following default parameters [19]. The genome and plasmid sequences were annotated using DFAST (https://dfast.nig.ac.jp).

Multilocus sequence typing analysis was performed to determine the sequence type according to the PubMLST protocol and database (https://pubmlst.org/organisms/escherichia-spp). The plasmid replicon type and ARGs were detected using PlasmidFinder v2.1 and ResFinder v4.1, respectively, with default parameters, on the CGE server (http://www.genomicepidemiology.org). Mobile gene elements were detected using blast with the ISfinder database (https://github.com/ thanhleviet/ISfinder-sequences). We compared individual contigs using progressiveMauve [9]. Linear comparison of the sequence alignment was performed using blast and visualised using Easyfig v2.2.2 (https://mjsull.github.io/Easyfig/). A circular representation of the plasmid was visualized and compared using blast Ring Image Generator 0.95 (http://brig.sourceforge.net).

## Results

One *mcr-9*-positive *E. coli* strain, Ec387, which had no other *mcr* genes, was detected among the 120 *E. coli* strains isolated in 2012 from diseased pigs with post-weaning diarrhoea (Table 1), representing a 0.8 % detection rate. However, *mcr-6*, *-7*, *-8* and *-10* were not detected in any of the tested strains. Ec387 was susceptible to colistin (MIC: 1 mg l^−1^), and resistant to ampicillin, cefazolin, kanamycin, tetracycline, nalidixic acid, ciprofloxacin, chloramphenicol and trimethoprim (Table 2). The *mcr-9*-harbouring transconjugants were obtained using selective agars supplemented with rifampicin, ampicillin, kanamycin, tetracycline, chloramphenicol or trimethoprim. *mcr-9*-harbouring transconjugants were not obtained using selective agar supplemented with rifampicin and colistin. All *mcr-9*-harbouring transconjugants were resistant to ampicillin, kanamycin, tetracycline, chloramphenicol and trimethoprim, but susceptible to colistin (Table 2).

**Table 2.** Antimicrobial phenotypes of the *mcr-9*-harbouring *Escherichia coli* strain

MIC* (mg l^−1^)	Donor	Recipient	Transconjugant
	Ec387	ML4909	TC_Ec387
Colistin	1	0.5	0.25
Ampicilin	**>128**	2	**>128**
Cefazolin	**8**	<1	4
Cefotaxime	<0.5	<0.5	<0.5
Gentamicin	1	<0.5	1
Kanamycin	**>64**	4	**>64**
Tetracycline	**>16**	2	**>16**
Nalidix acid	**>32**	4	4
Ciprofloxacin	**>4**	<0.03	<0.03
Chloramphenicol	**>32**	8	**>32**
Trimethoprim	**>8**	0.5	**>8**

*Bold type indicates resistant.

**Fig. 1.**
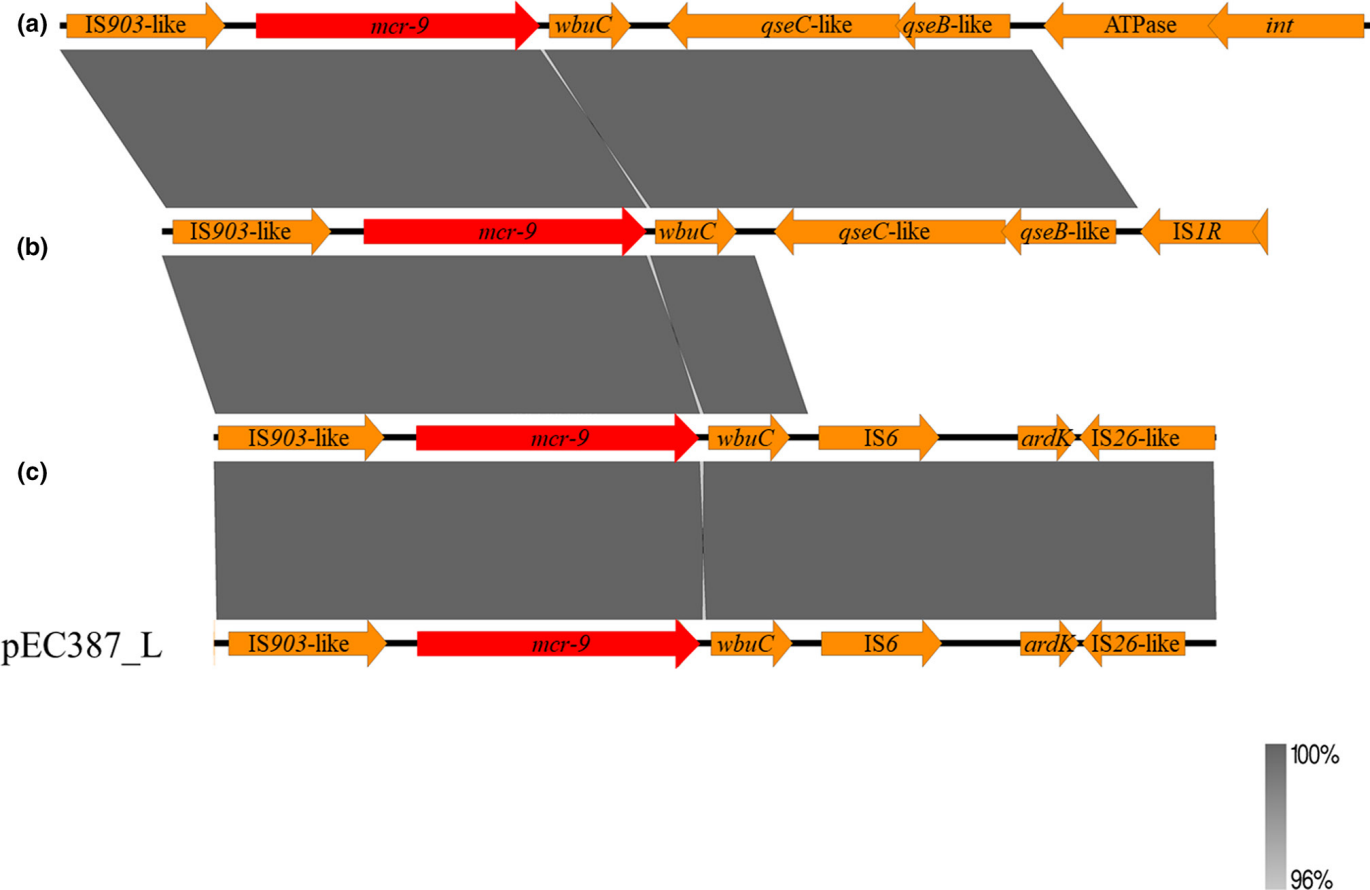
Linear comparison of regions surrounding *mcr-9* located in plasmid p387_L with (a) CP031102.1, (b) CP026661.1 and (c) CP020529.1. Red colour indicates antimicrobial resistance genes.

Whole-genome sequencing analysis revealed that Ec387 belonged to ST1196 and carried two plasmids: p387_L and p387_2 [accession no. AP024582 (chromosome), AP024583 (p387_L), and AP024584 (p387_2)]. P387_L was a 29 1362 bp plasmid classified as IncHI2/HI2A, which carried *mcr-9*, *bla*_TEM-1B_, *aph(3')-Ia*, *aph(3'')-Ib*, *aph(6)-Id*, *aadA2b*, *tetD*, *catA2*, *floR*, *sul1*, *sul2*, and *dfrA19*. p387_2 was an 88 903 bp plasmid classified as IncFIA/K/X1, which carried the *bla*_TEM-1B_, *aadA1*, *aadA2*, *aph(3'')-Ib*, *aph(6)-Ib*, *tetM*, *cmlA*, *floR*, *sul3*, and *dfrA12*. The region surrounding *mcr-9* in Ec387 was flanked by *IS903*-like and *IS26*-like mobile elements, but the *qseBC*-like element was not observed (Fig. 1).

**Fig. 2.**
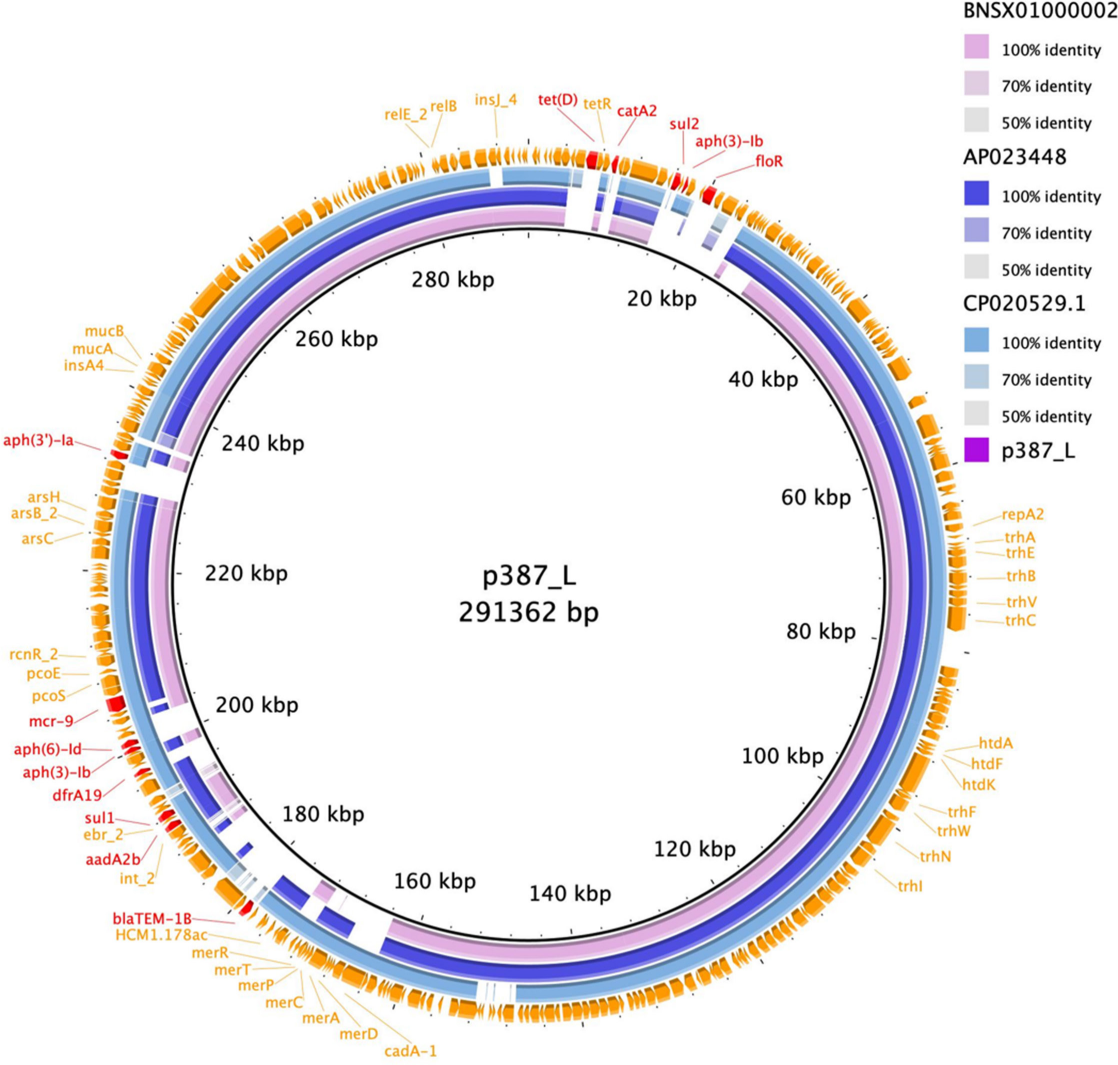
Comparison of *mcr-9-*harbouring IncHI2/HI2A plasmid p387_L with BNSX01000002, AP023448, and CP020529.1. Red colour indicates antimicrobial resistance genes.

Overall comparison of the plasmids showed that p387_L was highly similar to the InHI2/HI2A plasmid CP020529.1, which was isolated in the USA, and also similar to plasmids isolated in Japan (accession no. BNSX01000002 and AP023448) (Fig. 2). In addition, the p387_plasmid region harbouring ARGs, including *mcr-9*, was highly similar to that of the *Klebsiella pneumoniae* chromosome harbouring *mcr-9* isolated in Japan (accession no. BNSV01000001) (Fig. S1, available with the online version of this article).

## Discussion

In the present study, one *mcr-9*-positive colistin-susceptible *E. coli* strain (0.8 %) was detected among *E. coli* strains (*n*=120) derived from diseased pigs in Japan. Our previous study on these strains demonstrated the presence of the majority of *mcr* genes (*mcr-1*, *-3* and *-5*) in colistin-resistant strains, with a small number also detected in colistin-susceptible strains [3]. The expression of *mcr-9* can be induced in the presence of colistin when it is located upstream of *qseBC*, which was not detected in p387_L of strain Ec387 [20]. *mcr-9* has been detected worldwide, including in Japan, and a high prevalence of the gene was reported in colistin-susceptible *Salmonella* isolated from turkeys in the USA [5, 8, 21]. Generally, studies investigating the prevalence of ARGs in isolates first analyse their susceptibility to antimicrobials, and then investigate ARGs in the resistant isolates. Additional resistance mechanisms, including chromosomal mutation related to antimicrobial resistance, were investigated in resistant isolates in which the ARGs were not detected. However, in some cases, important ARGs, such as *mcr*, can be overlooked if only resistant isolates are analysed, which poses a risk of silent dissemination.

*mcr-9*-harbouring IncH12/HI2A plasmids also carried other ARGs, which could be transferred under the selection of antimicrobials without colistin. Globally, and in Japan, *mcr-9-*harbouring IncHI2/HI2A plasmids have been detected in Enterobacterales derived from humans and livestock. In some cases, these plasmids carry extended-spectrum β-lactamase- and carbapenemase-producing genes [9, 21, 22]. These results suggest that *mcr-9* can persist even when colistin is not used, through specific plasmids along with various other types of ARGs. Dissemination of these plasmids can propagate multidrug resistance and substantially restrict the choice of antimicrobials for treating bacterial infectious diseases.

Similarly, regions harbouring *mcr-9* and several other types of ARGs were previously confirmed in the plasmid p387_L and in the chromosome of *K. pneumoniae* isolated in Japan (accession no. BNSV01000001) [21]. Moreover, *mcr-9* located upstream of *qseBC* was detected in the chromosome of *Enterobacter* spp. [21]. These results suggest that *mcr-9* is transferred to various bacterial species and inserted into their chromosomes via a transposon.

## Conclusions

One *mcr-9*-harbouring colistin-susceptible *E. coli* strain was detected among 120 *E. coli* strains isolated from diseased pigs in Japan in 2012. The *mcr-9*-harbouring plasmid identified in the present study was similar to the previously reported global dissemination types. Therefore, surveillance of *mcr* genes, including among colistin-susceptible strains, is essential to accurately determine the prevalence of *mcr* genes.

## Supplementary Data

Supplementary material 1
