# Supplementary figures and images for: Conjugative IncHI2/HI2A plasmids harbouring mcr-9 in colistin-susceptible Escherichia coli isolated from diseased pigs in Japan
Source: Access Microbiol. 2022 Nov 28;4(11):acmi000454. doi: 10.1099/acmi.0.000454 (PMC9833416; doi:10.1099/acmi.0.000454)

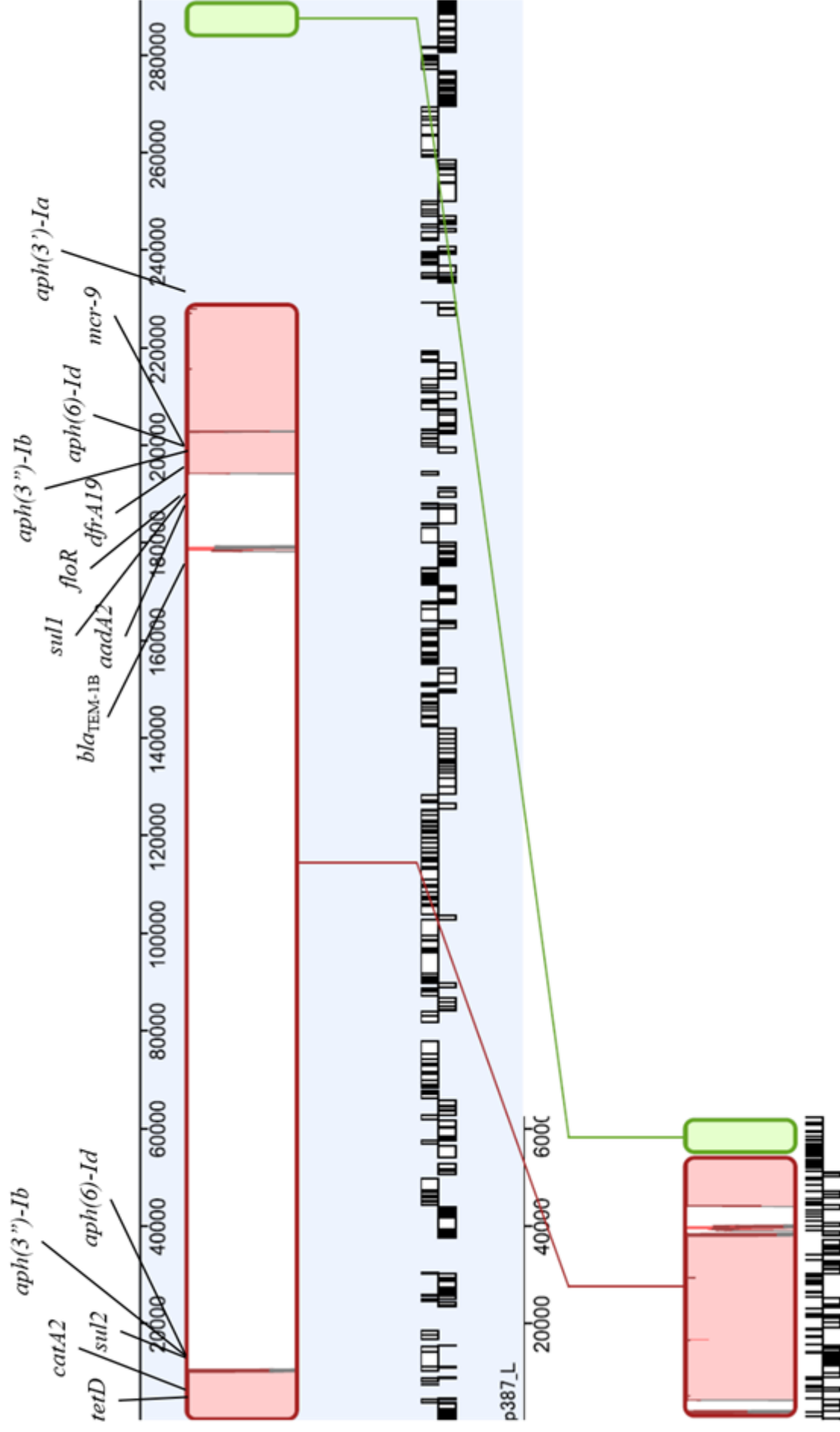

BNSV01000001

**Figure S1**

Supplement: Supplementary material 1 [file acmi-4-454-s001.pdf]
